# Community-Based Participatory Research: Lessons Learned from the Centers for Children’s Environmental Health and Disease Prevention Research

**DOI:** 10.1289/ehp.7675

**Published:** 2005-06-24

**Authors:** Barbara A. Israel, Edith A. Parker, Zachary Rowe, Alicia Salvatore, Meredith Minkler, Jesús López, Arlene Butz, Adrian Mosley, Lucretia Coates, George Lambert, Paul A. Potito, Barbara Brenner, Maribel Rivera, Harry Romero, Beti Thompson, Gloria Coronado, Sandy Halstead

**Affiliations:** 1 University of Michigan School of Public Health, Ann Arbor, Michigan, USA; 2 Friends of Parkside, Detroit, Michigan, USA; 3 University of California at Berkeley, School of Public Health, Berkeley, California, USA; 4 California Rural Legal Assistance, Salinas, California, USA; 5 Department of General Pediatrics, Johns Hopkins University School of Medicine, Baltimore, Maryland, USA; 6 Community Advisory Board member, Office of Community Health, Baltimore, Maryland, USA; 7 Principal, Dr. Bernard Harris Sr. Elementary School, President of Community Advisory Board, Baltimore, Maryland, USA; 8 Center for Childhood Neurotoxicology and Exposure Assessment, Robert Wood Johnson Medical School, University of Medicine and Dentistry of New Jersey, Piscataway, New Jersey, USA; 9 Executive Director, New Jersey Center for Outreach and Services for the Autism Community (COSAC), Ewing, New Jersey, USA; 10 Mount Sinai Center for Children’s Environmental Health and Disease Prevention Research, Department of Community and Preventive Medicine, Mount Sinai School of Medicine, New York, New York, USA; 11 Boriken Neighborhood Health Center, New York, New York, USA; 12 Fred Hutchinson Cancer Research Center, University of Washington, Seattle, Washington, USA; 13 U.S. Environmental Protection Agency Region 10, Prosser, Washington, USA

**Keywords:** children’s health, collaborative research, community-based participatory research, partnership

## Abstract

Over the past several decades there has been growing evidence of the increase in incidence rates, morbidity, and mortality for a number of health problems experienced by children. The causation and aggravation of these problems are complex and multifactorial. The burden of these health problems and environmental exposures is borne disproportionately by children from low-income communities and communities of color. Researchers and funding institutions have called for increased attention to the complex issues that affect the health of children living in marginalized communities—and communities more broadly—and have suggested greater community involvement in processes that shape research and intervention approaches, for example, through community-based participatory research (CBPR) partnerships among academic, health services, public health, and community-based organizations. Centers for Children’s Environmental Health and Disease Prevention Research (Children’s Centers) funded by the National Institute of Environmental Health Sciences and U.S. Environmental Protection Agency were required to include a CBPR project. The purpose of this article is to provide a definition and set of CBPR principles, to describe the rationale for and major benefits of using this approach, to draw on the experiences of six of the Children’s Centers in using CBPR, and to provide lessons learned and recommendations for how to successfully establish and maintain CBPR partnerships aimed at enhancing our understanding and addressing the multiple determinants of children’s health.

Over the past several decades there has been growing evidence of the increase in incidence rates, morbidity, and mortality for a number of health problems experienced by children—for example, asthma and other respiratory diseases (Landrigan et al. 2002; Mannino et al. 2002), developmental disabilities (Barone et al. 2000; Canfield et al. 2003), neuropsychologic disorders (Baldi et al. 2001; Schantz et al. 2003), and childhood cancers (Daniels et al. 1997). The causation and aggravation of these problems are complex and multifactorial, including genetic predisposition, demographic factors, psychosocial stressors, and environmental exposures. Numerous environmental exposures have been identified as contributing factors, including ambient levels of respirable particulate matter (Delfino et al. 2002; Eggleston 2000; Samet et al. 2000), ozone (Buchdahl et al. 2000; Mortimer et al. 2000), pesticides (Eskenazi et al. 2004; Landrigan et al. 2002; Perera et al. 2003), house dust mite and cockroach allergens (Litonjua et al. 2001; Sporik et al. 1999), and environmental tobacco smoke (Gergen et al. 1998; Gold 2000). The burden of these health problems and environmental exposures is borne disproportionately by children from low-income communities and communities of color (Evans and Kantrowitz 2002; Williams and Collins 1995). Recently, researchers and funding institutions have called for increased attention to the complex issues that affect the health of children living in marginalized communities (Schulz et al. 2002; Williams and Collins 1995), and communities more broadly, and have suggested greater community involvement in processes that shape research and intervention approaches, for example, through community-based participatory research (CBPR) partnerships among academic, health services, public health, and community-based organizations (CBOs) (Israel et al. 2003; Minkler and Wallerstein 2003; O’Fallon et al. 2000a). Each of the initial eight Centers for Children’s Environmental Health and Disease Prevention Research (Children’s Centers) funded by the National Institute of Environmental Health Sciences (NIEHS) and U.S. Environmental Protection Agency (EPA) was required to include a CBPR intervention project, and four additional Children’s Centers were subsequently funded (O’Fallon et al. 2000a). In all instances, the partners involved gained tremendous insights into how to conduct CBPR, and the challenges and benefits of using this approach to children’s environmental health research. The purpose of this article is to provide a definition and set of CBPR principles, to describe the rationale for and major benefits of using this approach particularly with environmental health research, to draw on the experiences of six of the Children’s Centers in using CBPR, and to provide lessons learned and recommendations for how to successfully establish and maintain partnerships aimed at enhancing our understanding and addressing the multiple determinants of children’s health.

## Definition and Principles of CBPR

### Definition of CBPR and community.

Within the field of public health, a number of partnership approaches to research have been called variously community-centered or community-based participatory/involved/collaborative research [for a review, see Israel et al. (1998)]. In addition, there is a large social science literature that has examined research approaches in which participants are actively involved in the process (e.g., Heron and Reason 2001; Jason et al. 2004; Kemmis and McTaggart 2000).

CBPR in public health is a partnership approach to research that equitably involves, for example, community members, organizational representatives, and researchers in all aspects of the research process, in which all partners contribute expertise and share decision making and responsibilities (Israel et al. 1998, 2003). The aim of CBPR is to increase knowledge and understanding of a given phenomenon and integrate the knowledge gained with interventions and policy change to improve the health and quality of life of community members (Israel et al. 1998, 2003). Within the context of CBPR, community is defined as a unit of identity. Units of identity refer to membership in, for example, a family, social network, or geographic neighborhood, and are socially created dimensions of identity (Steuart 1993). Community, as a unit of identity, is defined by a sense of identification and emotional connection to other members, common symbol systems, values and norms, shared interests, and commitment to meeting mutual needs (Steuart 1993). Communities of identity may be geographically bounded, for example, a neighborhood, or may be geographically dispersed, sharing a common identity (e.g., ethnic group, gays and lesbians). A city, town, or geographic area may include multiple overlapping communities of identity or may be an aggregate of individuals who do not have a common identity.

### Principles of CBPR.

Based on an extensive review of the literature, Israel et al. (2003) have identified a list of nine principles or characteristics of CBPR: CBPR recognizes community as a unit of identify; builds on strengths and resources within the community; facilitates a collaborative, equitable partnership in all phases of the research, involving an empowering and power-sharing process that attends to social inequalities; fosters co-learning and capacity building among all partners; integrates and achieves a balance between knowledge generation and intervention for mutual benefit of all partners; focuses on the local relevance of public health problems and ecologic perspectives that recognize and attend to the multiple determinants of health; involves systems development using a cyclical and iterative process; disseminates results to all partners and involves them in the dissemination process; and involves a long-term process and commitment to sustainability. There is no one set of principles that will be applicable for all partnerships; rather, all partnerships need to jointly decide what their core values and guiding principles will be, drawing on those presented here, as appropriate. These principles can be considered to be on a continuum, with those listed here being an ideal goal to strive for (Green et al. 2003; Israel et al. 2003).

## Benefits/Rationale for Using a CBPR Approach

As discussed in the literature, there are numerous benefits gained from using a CBPR approach (Israel et al. 1998; O’Fallon et al. 2000b). As reviewed elsewhere (Israel et al. 1998), among the key benefits are that it *a*) ensures that the research topic comes from, or reflects, a major concern of the local community; *b*) enhances the relevance and application of the research data by all partners involved; *c*) brings together partners with different skills, knowledge, and expertise to address complex problems; *d*) enhances the quality, validity, sensitivity, and practicality of research by involving the local knowledge of the participants; *e*) extends the likelihood of overcoming the distrust of research by communities that traditionally have been the “subjects” of such research; and *f* ) aims to improve health and well-being of the involved communities.

## Overview of the Children’s Centers’ CBPR Partnerships

To better understand the key issues in establishing and maintaining CBPR partnerships based on the experiences of six of the Children’s Centers, in this section we provide a brief description of the community context and structure of community involvement in these centers. Each of the 12 Children’s Centers was invited to participate in the development of this article. Because of time constraints, 6 of the 12 centers were not able to participate. Therefore, the experiences and lessons learned discussed here represent the efforts of the six Children’s Centers described below. The methodology used in writing this article included identification of academic and community partners from each of the six centers to be co-authors; conduct of several conference calls involving co-authors from each of the Children’s Centers to determine major topic areas to be covered; preparation by each center of a written mini-case study covering the topics outlined by the co-authors (based on ongoing conversations and documentation within the respective partnerships and, in some instances, a more formal evaluation of the partnership); synthesis and integration written by the lead author of the strategies, lessons learned, and recommendations discussed in the case studies; and review of the manuscript and revisions made based on the input and perspectives of the co-authors across the six centers.

### California Center for Children’s Environmental Health Research at the University of California, Berkeley (California/Salinas center).

Involving the predominantly Latino farmworker community in Salinas Valley, California, the California/Salinas center is a research partnership among the University of California at Berkeley, several state and federal agencies (the California Department of Health Services, the California Environmental Protection Agency, the Centers for Disease Control and Prevention), educational and research institutions (e.g., Stanford University, Battelle Laboratories), and numerous community agencies. The community partners, all within the state’s Salinas Valley in Monterey County, include Clínica de Salud del Valle de Salinas, Natividad Medical Center, South County Outreach Effort, California Rural Legal Assistance, the Grower-Shipper Association of Central California, and the Monterey County Health Department. The overall role of the partners is to advise center researchers in the development, implementation, analysis, and dissemination of culturally appropriate children’s environmental health research in the Salinas Valley.

The center has two advisory boards in the community, a community advisory board (CAB), which advises on all center studies, and an Intervention Farmworker Council (IFC), which was formed specifically to participate in the development and analysis of the intervention study. All partner organizations attend CAB meetings; however, the formal eight-member board itself is composed of representatives from three partner organizations and representatives of four additional organizations: the Monterey County Farm Bureau, the Monterey County Agricultural Commission, the Monterey County Board of Supervisors, and the California Assembly District 28. A representative from the seven-member IFC also sits on the CAB.

### Maryland Center for Childhood Asthma in the Urban Environment, Johns Hopkins University (Maryland center).

Involving the primarily low-income African-American community in East Baltimore, Maryland, the Center for Childhood Asthma in the Urban Environment recruited community members to join a CAB. The CAB met monthly with the study team based at the Johns Hopkins University School of Medicine and Bloomberg School of Public Health. Separate meetings by the CAB were also held. Members of the CAB included two school principals, a pastor, a nun assigned to work in the community, two community association presidents, a parent of a child with asthma, health personnel who had worked in the community, and a clinical social worker. The role of the CAB was to provide community input to the research investigators regarding the construction of the control group, recruitment strategy, and data collection to ensure participants received benefit from their involvement in the study.

### Michigan Center for the Environment and Children’s Health (Michigan center).

Involving a low-income, predominantly African-American community on the east side, and a low-income largely Latino community in Southwest Detroit, Michigan, the Michigan center is a CBPR partnership governed by a set of CBPR principles (Israel et al. 2001; Parker et al. 2003; Schulz et al. 1998). Community partners have been involved in all aspects and projects of the Michigan center, but they have been most involved with the Community Action Against Asthma (CAAA) research projects. The work of CAAA is guided by a steering committee (SC) composed of representatives from all of the partner organizations: the Detroit Department of Health and Wellness Promotion, the University of Michigan Schools of Public Health and Medicine, the Henry Ford Health System, and seven CBOs: Community Health and Social Services Center, Friends of Parkside, Warren-Conner Development Coalition, Latino Family Services, United Housing Coalition, Detroiters Working for Environmental Justice, and Detroit Hispanic Development Corporation. The SC has been actively involved in all major phases of the research and intervention, including the initial definition of the research questions, the design of all survey instruments, the hiring of key staff, the decision making on how to enroll and retain families in the intervention and study, and the interpretation, dissemination, and translation of research findings.

### New Jersey Center for Childhood Neurotoxicology and Exposure Assessment, University of Medicine and Dentistry, New Jersey (New Jersey center).

The community involved in the New Jersey center is the autism community of New Jersey, New York, Pennsylvania, and Connecticut. Drawing from a well-developed and extensive network of autism-based advocacy, support, and research-oriented groups, community groups have been involved with the center from the start. The community-based group Community Outreach and Support of the Autism Community, which is in its 39th year of operation with 4,000 members, is one of the center’s main partners. The partnership involves the Autism Schools, Edens Family of Services, and Douglass Developmental Center of Rutgers University. The partners work with the center on developing the hypothesis, the protocol design, recruitment, outreach, and communications with the autism community of the states involved. The partners participate in, coordinate, and moderate the town meetings the center has with the autism community of New Jersey and other states. Partners are also on the external advisory board of the center.

### New York Mount Sinai Center for Children’s Environmental Health and Disease Prevention Research (New York/East Harlem center).

Involving the predominantly African-American and Latino communities in East Harlem, located in northern Manhattan, New York, the New York/East Harlem center formed partnerships among the center’s principal investigators (PIs) and the leadership of two federally qualified community health centers (Boriken Neighborhood Health Center and Settlement Health). Both health centers are governed by boards whose members represent health care consumers and community residents. Medical school investigators and the community partners agreed from the onset that joint decision making and collaboration was needed to design the intervention and research protocols, select and hire field staff, provide oversight to field staff in study recruitment and conduct of the intervention, organize and sustain a CAB, and disseminate information and lessons learned to the local community and to policy makers. An SC composed of the executive director and/or associate director of the health center, a health center physician, the PI, and the project research coordinator was set up at each health center; representatives from the community partner sites attended monthly center meetings at the Mount Sinai School of Medicine.

A CAB composed of 20 active community stakeholders was formed and met semiannually to advise the researchers on dissemination of information and to help design broader community interventions intended to change both individual and institutional behaviors related to pesticide use and pest control. Members included tenant association leaders and members, housing managers, school teachers, parent association leaders, social service agencies, community health providers, and local elected officials.

### Washington Center for Child Environmental Health Risks at the University of Washington (Washington center).

Involving the predominantly Hispanic farmworker community in 16 small towns and eight labor camps in lower Yakima Valley of eastern Washington State, the Washington center’s community project is a partnership composed of a variety of CBOs and individuals. Examples of such groups include the local farmworkers’ union, local farmworkers’ clinics, local department of agriculture, State Department of Health, Department of Labor and Industries, U.S. EPA district 10, Washington Growers’ League, farmworker advocates, farmworkers, health care providers, legal representatives, local newspapers, a Spanish-speaking radio station, and university extension offices. The partnership has been formalized into an 18-member CAB that is facilitated by a project coordinator hired from the community and by the CAB. Rules of the partnership emphasize interaction, respect, and the principle that all ideas are freely expressed and discussed. The CAB has been involved in the community project from the beginning, in a number of areas, including providing information regarding the concerns among local residents about pesticide exposure; participating in the design of the data-collection content and procedures, intervention design, recruitment and implementation, publication, and dissemination; and hiring of local staff. A member of the CAB also serves on the center’s external advisory committee.

## Key Issues in Establishing and Maintaining CBPR Partnerships: Strategies, Lessons Learned, and Recommendations

### Key Components of the Children’s Centers CBPR Partnerships

In keeping with the principles of CBPR listed above, a number of components or dimensions can be incorporated into CBPR partnerships. Table 1 provides a brief picture of how each of the Children’s Centers has addressed these components. An elaboration and analysis of some of these major components, lessons learned, and recommendations for conducting CBPR, based on the experiences of the centers, is provided below.

### Definition of Community and Identification and Selection of Community Partners

A critical consideration in establishing a CBPR partnership is deciding how the “community” is defined, who represents the “community,” and how partners are selected (Israel et al. 2003; Koné et al. 2000).

#### Diverse approaches to definitions of community.

All but one of the Children’s Centers defined the community(ies) involved using geographic boundaries and common characteristics. In urban areas these were more neighborhood based (e.g., East Baltimore, East Harlem), whereas in rural areas the geographic boundaries were more spread out and included multiple small towns. Within each of these geographic communities, there were similar demographic and other characteristics (e.g., predominantly low income, African American, Latino, farmworkers). In addition, all of the communities experienced high incidence and prevalence of the particular environmental issue and/or health problem(s) that were the focus of the overall center. Furthermore, all of the communities have considerable strengths and assets (e.g., social networks, community organizations). Some of the centers involved smaller “communities of identity” (Steuart 1993), as defined above, such as a predominantly African-American neighborhood. Some of the Children’s Centers defined the community as a larger geographic area in which all of the stakeholders needed to be involved. For example, in the California/Salinas and Washington centers, both farmworkers and representatives from agricultural industry organizations were invited to participate. The New Jersey center defined the community as one that has children with autism and involves partners and participants from ethnically and economically diverse groups across several states.

One of the key principles of CBPR is that it recognizes community as a unit of identity and seeks to identify and work with existing communities of identity (Israel et al. 1998, 2003). This approach acknowledges that communities of identity have numerous individual and organizational skills and resources, but that they may also benefit from external skills and resources. Thus, CBPR partnerships may involve individuals and groups that are not members of the community of identity (Israel et al. 2003). For example, although a group of farmworkers might be most appropriately conceptualized as a community of identity for a CBPR effort, there may be some advantages of also including representatives from the agricultural industry, such as their potential role in policy change. When establishing a partnership, it is important to examine the advantages and disadvantages of extending membership beyond the community of identity at the outset. In the farm-worker community example, given the power differentials that exist between farmworkers and growers, the economic dependence of farmworkers, and the history of adversarial relations, it is critical to determine whether farmworkers will be comfortable expressing their opinions and whether their voice will be heard if growers are also at the table. One possible strategy is to start with the most immediate community of identity, that is, farmworkers, and after trust is established, and with their concurrence, bring additional parties into the process. Another strategy, used by the California/Salinas center, was to establish a separate group, the IFC, composed primarily of farmworkers, that was actively involved in the design and implementation of the intervention component of their center.

#### Different strategies for selection and identification of partners.

Several different strategies were used in the selection and identification of potential partners. A key aspect of several of the Children’s Centers’ approaches was building on prior positive working relationships that existed between academia and the communities involved. For example, the identification of community partners for the New York/East Harlem center was an outgrowth of > 25 years of collaboration between the academic and primary health center partners involved.

Similarly, the Michigan center evolved from an already existing community–academic partnership, the Detroit Community-Academic Urban Research Center (URC) (Israel et al. 2001; Lantz et al. 2001). In 1997, the URC board identified childhood problems related to the environment (e.g., asthma) as a priority area for future research and interventions, and subsequently when the request for proposals for the Children’s Centers was released, the URC board decided to apply. The Michigan center involves many of the original URC academic and community partners as well as new researchers and community organizations with expertise in asthma and/or the environment. Using a somewhat different approach, the New Jersey center selected autism advocacy groups or schools for children with autism that are regionally and nationally recognized by the autism community.

Another viable strategy for identifying and selecting partners is to conduct a community analysis to assess the values, needs, resources, barriers, and facilitators required for community action around an issue (Eng and Blanchard 1990–1991; Thompson et al. 2001). The Washington center conducted a community analysis to gain an increased understanding of the positions of the major participants or groups and to find common ground among the various parties involved. The results indicated a number of common themes as well as a wide disparity among groups in their views on pesticides. These were discussed with a community planning group, which recommended that because of the contention around pesticides, every constituent should be invited to participate in decision making (for more details, see Thompson et al. 2001).

Another consideration in selecting organizations as partners in a CBPR project is identifying who will represent the organization. To the extent possible, individuals who participate on CBPR boards need to be in leadership positions or have the authority to make decisions without always having to ask the leadership. At minimum, they need to have easy access to and the active and visible support of their organization’s leadership (Israel et al. 2001). Although in many instances it is ideal to have top leadership directly involved, such leaders are often constrained by other demands on their time and may be less able to actively participate. Another viable strategy is to have a designated representative and an alternate, with the alternate receiving all mailings and communications and attending meetings when the primary member can not.

### Overall Role of Community Partners in CBPR Projects

One of the key concepts in conducting CBPR is the role of participation of the community members and researchers (Wallerstein and Duran 2003). Some of the core questions that need to be addressed include the following: What aspects of the CBPR process are community partners participating in? What level of influence or control do they have over the decisions made? What level of commitment do university partners have to creating an equitable partnership that attends to power differentials? There are a number of different ways in which community participation has been conceptualized, with the major similarity across these different perspectives being the concept of a continuum of control or power, ranging from the low end of the spectrum, where community members serve on advisory boards and have some limited involvement but little influence and control over the project, to the other end, where community members have full control over all aspects of the research process (Arnstein 1969; Balcazar et al. 2004). Not all CBPR partnerships will achieve the same level of community participation.

As shown in Table 1, four of the Children’s Centers have CABs (California/Salinas, Maryland, New York/East Harlem, and Washington) composed of representatives from highly diverse organizations. In most instances, the researchers and staff are not considered members of the CAB, although they frequently attend CAB meetings. Although the same “CAB” name is used across these four centers, the frequency of meetings, purpose of the CAB, and degree of community participation and control differ considerably and, in some instances, have changed over time. (See Table 1 for information on the frequency of meetings and facilitation of meetings.)

The experience of the Maryland center’s CAB shows how the role of community partners evolved over time. The partnership initially functioned to review study protocols and patient education material and assist in defining the target community. The CAB, composed of 10–14 members, was strictly advisory in nature and functioned within a limited sphere. The CAB expressed concerns about its role as being either too limited or too ambiguous because their opinions and input did not appear to influence the work of the research team. With the guidance of the CAB president, several strategies were developed (e.g., educational session, community tour, retreat) to assess the partnership and enhance the working relationships to mutually satisfying levels so that all could benefit. Through this process, the foundation was laid for increased collaboration and establishment of a shared culture. The CAB moved from “advisory” toward sharing “governance” of the project.

The Michigan center provides an example of another approach to organizing a CBPR partnership. The center is guided by an SC composed of representatives from academia, CBOs, and public health and health care institutions and one community member-at-large. The SC members were identified when the grant proposal was being written, with the size ranging from 14 to 17 members over the 5-year project period, and it has met monthly since the center was established. The meetings are co-facilitated by university faculty members at the initial request of the SC.

### Role of Community Partners in Specific Stages of the Research Process

Community participation in and influence over each of the areas listed in Table 1 are considered to be a critical component of CBPR partnerships (Israel et al. 1998, 2003; Minkler and Wallerstein 2003). Ideally, any CBPR project involves community partners from the beginning stages, including defining the initial research priorities and questions. In responding to a call for proposals, this requires that either a partnership already exists or that time and resources be available to bring potential partners together to decide on these key issues. Unfortunately, this is often not the case, and researchers may have to approach potential community partners after decisions have already been made regarding the research priorities. It is important to identify partners who share an interest in the priorities selected, and considerable opportunity needs to be provided for input and decision making in subsequent stages of the research.

All of the Children’s Centers actively involved their community partners and greatly benefited from their participation in the design and implementation of the intervention research studies. Community partners can be instrumental in the overall study design. For example, the Maryland center CAB members voiced their concern that each participant be treated the same and receive immediate benefit from their participation, and under their advisement, the investigators changed the control group to a “treat later” group to ensure that all participants received the intervention. Community partners also provide valuable suggestions for specific intervention strategies—for example, a calendar contest in the schools.

Each of the Children’s Centers has greatly benefited from their community partners’ role in the development and implementation of data collection instruments. For example, the involvement of community partners and local staff in meetings and focus group interviews has provided information that resulted in more complete data collection and investigation of areas initially not included by the researchers, including both content and cultural appropriateness of language and methods (Edgren et al. 2005).

The community partners and local staff across all the Children’s Centers have played an active role in the design and implementation of recruitment and retention activities. Their input has been a significant factor in ensuring cultural and linguistic appropriateness and effectiveness in all written materials as well as in understanding the social, economic, political, and housing conditions in the communities involved that have an impact on participant involvement.

In some of the Children’s Centers the community partners were actively involved in guiding data collection activities. In particular, the hiring and training of local community members as data collectors provide the trust needed between the data collectors and respondents to enhance the quality and validity of the data.

The analysis and interpretation of data are areas in which community partners frequently have limited involvement. None of the Children’s Centers involved their community partners directly in data analysis. Given the time demands and technical aspects of data analysis, the lack of community involvement may be most appropriate. However, this may be an area in which community partners are interested in enhancing their skills, and thus, this needs to be discussed among the partners (Israel et al. 2003). What is crucial for all CBPR efforts is that the results of data analyses be fed back to the partners in ways that are understandable, and that the partners engage in a process of interpreting the data (Israel et al. 2003). Community partners are able to provide meaning to results that outside researchers may not have considered, for example, insights into the role of cultural dynamics and other contextual factors. The involvement of community partners in the interpretation of findings also has helped to increase community partners’ knowledge and comfort with research data and results. This has enabled all partners to share more equally in presenting results to study participants and in other settings.

As depicted in Table 1, there are a number of different ways in which community partners are involved in the dissemination of study findings—for example, presentations at meetings, publications, information booklets, newsletters, and radio announcements. Community partners should have the opportunity to be involved as co-authors and co-presenters on publications and presentations, to the extent that they are interested. Researchers need to recognize, however, that obtaining community partner involvement in this regard may require strategies such as face-to-face meetings and discussions of drafts rather than merely sharing written documents and expecting a written response.

To develop and maintain an effective CBPR partnership, and to increase understanding of the factors that contribute to successful partnerships, it is necessary to evaluate the CBPR partnership process, for example, to assess the extent to which and ways in which CBPR principles are followed (Israel et al. 2001, 2003; Lantz et al. 2001; Parker et al. 2003). Such an evaluation can include quantitative and qualitative methods and needs to involve all partners in the process and include regular feedback of results to make changes in how the partnership functions, as needed (Israel et al. 2003; Lantz et al. 2001; Parker et al. 2003; Schulz et al. 2003) (see, e.g., the evaluation conducted at the Michigan center by Parker et al. 2003).

### Group Processes Involved

In keeping with the key principles of CBPR, it is critical that every partnership consider how it will strive to achieve shared equity, influence, and control over the decision-making process (Israel et al. 1998, 2003). This requires devoting considerable time and attention to the group’s process (Becker et al. 2005), which may be frustrating for some partners if it is perceived as taking time and resources away from the accomplishments of specific objectives (Israel et al. 2001, 2003; Lantz et al. 2001). A number of characteristics of effective groups are presented in the literature, such as two-way communication, appropriate decision-making procedures, shared power, the ability to resolve conflicts constructively, and the ability to engage the expertise of all members (Johnson and Johnson 2003). The extent to which CBPR partnerships pay attention to group dynamics and achieve these characteristics (i.e., process objectives) has implications for the group’s ability to achieve its short- and long-term goals (i.e., impact and outcome objectives) (Schulz et al. 2003).

The establishment by a partnership of operating norms and procedures that are in accordance with and reinforce the key principles of CBPR (Israel et al. 1998) is a key factor that attends to group dynamics issues through facilitating the trust and relationship building necessary to successfully conduct CBPR. These need to be consistent with the characteristics of effective groups mentioned above (Johnson and Johnson 2003) and to promote understanding and demonstrate competence in working with diverse cultures, for example, regarding class, gender, ethnicity, age, and sexual orientation (Israel et al. 1998). These norms and procedures need to be identified and agreed on by all the partners involved, documented in writing (they do not need to be as formal as by-laws, although they can be), and reviewed periodically to assess the extent to which they are being followed (Israel et al. 1998, 2001).

There is also considerable emphasis in the literature on the value of partnerships jointly developing overarching CBPR principles or core values (Israel et al. 2001), which also helps attend to group dynamics issues. The Maryland center CAB spent several CAB meetings to identify, define, and adopt its core values, which include cultural competence and inclusiveness, meaning that partners recognize, accept, and celebrate their differences and community perspectives are included and valued; and effective and open communication among partners including recognition of participants’ right to know study findings. The New York/East Harlem center’s guiding principles for shared decision making and power sharing between the research institution and the health centers include joint selection of field staff with an emphasis on hiring from the community, and full review and agreement on research protocols, data collection instruments, recruitment and retention strategies, and educational materials. The California/Salinas center’s guiding principles include giving back more to the community than is taken, being culturally sensitive and appropriate, sharing decision making, and providing long-term and sustainable resources to the community.

### Compensation for Community Partners

As indicated in Table 1, a range of approaches were used by the Children’s Centers regarding compensating community partners for their involvement. The emphasis on equity as a key principle of CBPR underscores the importance of addressing this issue. The extent and amount of compensation need to be considered by each partnership in the context of the level of involvement (e.g., annual meetings compared with monthly meetings) and by type of organization (e.g., members from agricultural industry and health care systems, compared with farmworkers and CBOs). Although it may not be possible to fully compensate community partners monetarily for the time they contribute to the partnership, adequate recognition of and compensation for their contributions should be provided. In addition to providing direct financial resources and coverage of travel expenses, this could take the form of technical assistance and training. For example, in the New Jersey center, the community-based partners did not want to have any financial ties to the study to ensure their independence, although compensation did occur through the center’s provision of information and assistance with fund raising. The issue of equity can also be considered in terms of resources provided to the community at large—for example, hiring local community members and providing services such as health information at local health and work fairs in the community. The process for deciding how to handle compensation needs to be joint, open, and transparent.

### Staff Hired from the Local Community

Another key factor has been the establishment of field offices in the community, and the hiring of local community members as staff who are similar to the project participants (e.g., culture, language). Although setting up a field office is particularly important when the research institution is not located in the community in which the project is involved, it is also worth considering when the academic institution that is within the community is perceived as having limited access or being inhospitable. Across the Children’s Centers, the staff positions for which community members have been hired have included field coordinators, interviewers, other data collectors (e.g., air quality monitoring), and intervention staff (e.g., outreach workers). In some instances, local staff were hired as employees of a community partner organization, whereas in other cases local staff were hired as employees of the academic institution involved. Local staff have played a crucial role in all phases of the projects (e.g., providing feedback on study protocols and data collection instruments, and problem solving implementation issues that arise). Local staff in the California/Salinas, Maryland, Michigan, and Washington centers have been the day-to-day “face” of the project in the community and have provided a bridge among the researchers, community partners, intervention participants, and community members-at-large. This regular interaction has been crucial for building and maintaining the trust necessary to obtain the input needed to conduct culturally appropriate and high-quality CBPR projects. Although some local staff had prior experience working in research and interventions, in other instances relevant training was provided.

## Challenges of Using a CBPR Approach for Children’s Environmental Health Research

Some of the major challenges associated with using CBPR that were faced by the Children’s Centers are presented briefly below. Some strategies for overcoming these challenges are presented in the preceding section, and others are discussed further below in the context of overarching lessons learned.

### Costs incurred and lack of resources.

There are numerous costs for both community and academic partners involved in CBPR efforts and insufficient resources for overcoming them (Israel et al. 1998; Koné et al. 2000; Minkler 2004). An effective partnership requires time and infrastructure support, for example, to establish and maintain trust, attend meetings, jointly participate in all phases of the research, and foster capacity building. Community partner organizations face financial costs from involvement, such as lack of adequate reimbursement for their time spent participating, as well as opportunity costs for time taken away from other job responsibilities (Koné et al. 2000; Parker et al. 2003). Research investigators are also constrained by the time and costs required (Parker et al. 2003).

### Institutional constraints.

Many institutional constraints are faced in conducting CBPR (Israel et al. 1998). Among the challenges faced by the Children’s Centers are university institutional review board (IRB) processes that do not take into account the needs of CBPR projects (e.g., the need to be flexible and revise protocols based on community input), overhead issues, long delays associated with data analysis and returning results to the community, and hiring policies that require traditional job descriptions and educational degrees. Community partners, many of whom are not paid by the project and have numerous other professional responsibilities, may not be supported by their supervisors if their involvement is perceived to be taking time away from other organizational responsibilities.

### Lack of trust and respect: institutional history.

Building and maintaining trust both between the university and community as well as at times within community partners are a substantial challenge (Israel et al. 1998; Minkler 2004). For example, when diverse groups of stakeholders are brought together who have a long and adversarial history, such as those representing farmworker and agricultural industry interests, as was the case in California/Salinas, this can present serious difficulties for the partnership. Some key questions that need to be asked here include the following: Is the trust of the board being compromised by trying to bring too many interests to the table? Are CBPR partnerships the appropriate entity to try to bridge longstanding and political tensions that may exist? Does the participation of “all” stakeholders really promote the support of study results and the future translation of findings into policy?

### Ensuring community participation and influence.

Related to time constraints and costs, another challenge faced by CBPR partnerships is ensuring community participation and influence (Green and Mercer 2001; Israel et al. 1998; Minkler 2004). Community building is a very important and often overlooked step in building a “collaborative, equitable” partnership, which requires skill and takes time and commitment on the part of all partners to foster participation and shared decision making.

### Lack of training and experience in conducting CBPR.

Another challenge is that many researchers and community partners have limited training and experience in conducting CBPR. Although there is a large and growing literature on how to carry out CBPR efforts (Minkler and Wallerstein 2003), many researchers and community partners have not received direct training and have limited opportunity to engage in learning opportunities to strengthen their skills in this area. This is particularly challenging in situations such as the Children’s Centers, where community involvement was a requirement from the funding institutions, and not all researchers fully understood what the implications of that meant.

### Different emphasis on goals, values, priorities, and perspectives.

There are a number of areas where community and academic partners may differ in their emphasis on goals, values, priorities, and perspectives (Israel et al. 1998). For example, in several Children’s Centers, community partners were eager to implement the interventions and disseminate preliminary results, whereas researchers were concerned that the premature dissemination of results would contaminate study findings and lead to scientific criticism and consequences for publications and future funding. Challenges also occur given that members of partnerships have, for example, different values, beliefs, and cultures (Israel et al. 1998; Minkler 2004). Importantly, these various differences do not suggest a “right” or “wrong” way that partnerships should operate; rather, they suggest the need to consider and accommodate diverse perspectives.

### Different languages and styles of communication.

Another challenge is that members of CBPR partnerships speak different languages and use different styles of communication. One difference that several of the Children’s Centers faced was that most members speak English whereas some speak Spanish. This creates challenges in terms of conducting bilingual meetings, having all materials in Spanish as well as English, and ensuring participation from predominantly Spanish-speaking members. In addition, researchers often use scientific words and language that are not easily understandable, and community partners may use words and colloquialisms that scientists do not understand. Furthermore, researchers at the Children’s Centers often use electronic mail for communicating, frequently needing/expecting quick responses, and some community partners do not have jobs that enable them to be in such frequent email contact, and others do not use email at all.

## Overarching Lessons Learned and Recommendations

Throughout this article, we have shared the experiences of the Children’s Centers in using a CBPR approach and provided lessons learned and explicit as well as implicit recommendations for how to conduct CBPR. Building on these, in this section we present several overarching lessons learned and recommendations.

- Sufficient time, resources, and benefits are needed for all partners to ensure active and meaningful participation.
- Considerable commitment and time are needed to establish and maintain trust.
- Jointly developing and following operating norms and CBPR principles/core values are essential.
- Acknowledging and addressing power and equity issues are critical.
- Funding and academic institutions need to extend their criteria for research excellence and productivity (e.g., the randomized control trial in which one group receives no intervention may not always be feasible or desirable within a CBPR context) and be flexible to incorporate the input of community partners (e.g., IRB review and approval processes).
- Commitment to translating research findings into interventions and policies is of utmost importance.
- Hiring and training staff from the local community are essential.
- Recognizing, respecting, and embracing different cultures of the partners and partner organizations are imperative for successful CBPR efforts.

## Concluding Remarks

CBPR is an especially useful approach for working with marginalized communities that experience a disproportionate burden of environmental, health, and other problems and that typically have not been included in deciding what types of research and interventions are most appropriate for and likely to be most effective in their communities. Although it is neither possible nor appropriate to use CBPR in all research studies, other research approaches may benefit from incorporating some of the principles and strategies recommended throughout this article.

With the NIEHS and the U.S. EPA providing the notable exceptions, most organizations supporting health research, especially basic research (e.g., epidemiologic, genetic), do not require researchers to work with communities in the identification, design, implementation, analysis, and dissemination of research. The NIEHS/U.S. EPA’s emphasis on community–academic partnerships has encouraged researchers conducting health effects and exposure research, in addition to those conducting intervention research, to develop such partnerships and to orient their research in ways they previously had not. We hope that the experiences and benefits gained from these Children’s Centers’ partnerships will provide guidance and encouragement to the National Children’s Study and others to incorporate similar CBPR approaches to address environmental and children’s health issues.

## Figures and Tables

**Table 1:** Key components of Children’s Centers CBPR partnerships.

	Center location							
	California/Salinas^a^					New York/East Harlem^b^		
Component	CAB	IC	Maryland	Michigan	New Jersey	BNHC	SH	Washington
Intervention study design								
Group randomized controlled trial	X		X^c^		X	X		X
Randomized staggered controlled trial				X				
Intervention participants					X^d^			
Predominantly low income	X		X	X		X		X
African American			X	X		X		
Latino/Hispanic	X			X		X		X
White non-Hispanic								X
Partnership title								
CAB	X		X			X		X
SC				X				
Intervention council		X						
IPO					X			
Members/organizational representatives involved in CAB, SC, intervention council, and IPO								
Individual community members		X	X	X	X	X		X
CBOs	X		X	X	X	X		X
Faith-based organizations			X					X
Local health department	X			X		X		X
Community health center/health personnel	X		X	X	X	X		X
Hospitals/integrated care systems				X	X	X		
University				X	X	X		X
Other governmental agencies (e.g., schools, social service)	X		X	X	X	X		X
Business/industry	X					X		X
Others attend meetings (e.g., staff, faculty)	X	X	X	NA	X	X		X
Other organizations^e^	X					X		X
No. of board/committee members	8	7	10–14	14–17	5	20		18
Frequency of meetings								
Monthly				X	X	X^f^	X^g^	X^h^
Bimonthly		X	X					X^h^
Quarterly								X^h^
Semiannually	X^i^							X^h^
Annually					X			
Location of meetings								
Clinic/medical center in community	X				X	X	X	X
Rotate among community partner organizations				X	X			
Neighborhood school			X					
Facilitator of meetings								
Project staff					X	X		X
Researchers/faculty members	X			X	X			
Community members					X			
Staff and community member co-facilitate		X	X					
Role of community partners in different stages of research/activities								
Define initial research questions/priorities				X	X			X
Design/implementation of research/intervention	X	X	X	X	X	X	X	X
Development of data collection instruments/protocols	X	X	X	X	X	X		X
Hire staff	X			X		X	X	X
Recruitment of participants	X			X	X	X	X	X
Retention		X	X	X	X	X	X	NA
Review/comment educational and feedback materials		X	X	X	X	X	X	X
Data collection		X		X				X
Data analysis		X						
Data interpretation	X	X	X	X				
Dissemination								
Review/provide feedback	X				X			X
Scientific papers	X				X			X
Co-present professional meetings	X		X	X	X			
Co-present community forums/meetings	X		X	X	X			X
Co-author journal articles/book chapters	X			X		X		X
Review/comment newsletters/flyers	X	X	X	X	X	X	X	X
Input on website development				X	X			
Evaluation of partnership		X	X	X	X			
Development of additional research proposals/projects	X		X	X	X			X
Provide entrée/linkages with other community organizations	X		X	X	X			X
Group processes								
Operating norms/ground rules		X		X	X	X	X	X
CBPR/guiding principles/core values	X		X	X	X	X	X	X
Dissemination principles	X		X	X	X			X
Publication review protocol	X			NA	NA			
Community partner compensation for participation								
Honorarium to organizations				X				
Honorarium/reimbursement to individuals	X^j^	X	X	X				
Subcontract for services				X		X	X	X
Percent of administrative overhead						X	X	
No compensation	X^j^				X^k^			X
Communication outside of meetings								
Minutes		X	X	X	X			X
Mailings		X		X	X			X
E-mail	X		X	X	X			X
Fax	X		X	X	X			X
Telephone	X			X	X	X	X	X
In-person meetings	X			X	X	X	X	X
Staff hired from local community								
Field coordinator	X		X	X				X
Interviewers	X		X	X				X
Other data collectors (e.g., home inspection)	X		X	X				X
Intervention staff	X		X	X				X

Abbreviations: BNHC, Boriken Neighborhood Health Center; IC, intervention council; IPO, individual partner associations; SH, Settlement Health.

a Eight-member CAB developed after funding received to be involved in overall center activities. After 3 years, additional IFCs established to advise center on intervention-related activities.

b Two partnerships were established, one with BNHC at the beginning of the project, and one with SH at the end of the second year, both federally qualified community health centers. The information in this table applies primarily to these two partner organizations. In addition, a CAB composed of 20 active community stakeholders was established by the researchers and two partner organizations and meets semiannually to advise researchers on the translation of results and to provide feedback during the process of the study. Members of the CAB are indicated on the table, but additional information in the table does not apply to the role of the CAB.

c Over time, under advisement of CAB, control group changed to “treat later” group.

d The participants are approximately representative of the demographics of the states involved (i.e., New Jersey, New York, Pennsylvania, Connecticut).

e Examples of other organization members include legal assistance, farm bureau, and agricultural commission.

f Started with monthly meetings for the first 3 years. As recruitment and intervention phase ended, meetings became less frequent.

g Monthly meetings were recommended but did not occur. Most decisions were made by leaders of the partner organizations on an as-needed basis, via the telephone and face-to-face contact.

h Started with monthly meetings, after first year moved to bimonthly and subsequently quarterly, then semiannually.

i Meetings have been on an annual basis with additional feedback provided through subcommittee meetings and one-on-one communications. Meetings currently being conducted semiannually.

j Honorarium provided for one member who missed work time to attend annual meeting; other members were not compensated for their attendance.

k Members of the center actively participate in many activities of the community partners, including fund raising activities and multiple presentations to the community partners on topics such as autism, children’s development, and the effects of environmental exposure.
